# Clinical and Microbiological Characteristics of Heteroresistant and Vancomycin-Intermediate *Staphylococcus aureus* from Bloodstream Infections in a Brazilian Teaching Hospital

**DOI:** 10.1371/journal.pone.0160506

**Published:** 2016-08-30

**Authors:** Thaina Miranda da Costa, Priscylla Guimarães Migueres Morgado, Fernanda Sampaio Cavalcante, Andreia Paredes Damasco, Simone Aranha Nouér, Kátia Regina Netto dos Santos

**Affiliations:** 1 Laboratório de Infecção Hospitalar, Departamento de Microbiologia Médica, Instituto de Microbiologia Paulo de Góes, Universidade Federal do Rio de Janeiro, Rio de Janeiro, Brazil; 2 Hospital Universitário Clementino Fraga Filho, Faculdade de Medicina, Universidade Federal do Rio de Janeiro, Rio de Janeiro, Brazil; Rockefeller University, UNITED STATES

## Abstract

This study analyzed clinical and microbiological characteristics of heteroresistant (hVISA) and vancomycin-intermediate *Staphylococcus aureus* (VISA) from bloodstream infections (BSI) in a Brazilian teaching hospital, between 2011 and 2013. Minimum inhibitory concentrations (MIC) of antimicrobials were determined by broth microdilution method and SCC*mec* was detected by PCR. Isolates with a vancomycin MIC ≥ 2mg/L were cultured on BHI agar with 3, 4 or 6 mg/L (BHIa3, BHIa4 or BHIa6) of vancomycin and BHIa4 with casein (BHIa4ca). Macromethod Etest^®^ and Etest^®^ Glicopeptides Resistance Detection were also used. VISA and hVISA isolates were confirmed by the population analysis profile then typed by pulsed-field gel electrophoresis (PFGE) and multilocus sequence typing. Medical data from the patients were obtained from their medical records. Among 110 consecutive isolates, 31 (28%) were MRSA and carried the SCC*mec* type II (15 isolates) or IV (16 isolates). Vancomycin MIC_50_ and MIC_90_ were 1 and 2 mg/L, respectively. MRSA isolates had increased non-susceptibility to daptomycin (*p* = 0.0003). Six (5%) isolates were VISA, four of which were MRSA, three SCC*mec* type II/USA100/ST5 and one type IV/USA800/ST3192. One MRSA SCC*mec* II isolate grew on agar BHIa3, BHIa4 and BHIa4ca, and it was confirmed as hVISA. Among the six VISA isolates, five (83%) grew on BHIa3 and three (50%) on BHI4ca. Four of the six VISA isolates and the one hVISA isolate were from patients who had undergone dialysis. Thus, a possible dissemination of the SCC*mec* II/USA100/ST5 lineage may have occurred in the hospital comprising the VISA, hVISA and daptomycin non-susceptible *S*. *aureus* Brazilian isolates from health care associated bloodstream infections.

## Introduction

*Staphylococcus aureus* continues to be an important human pathogen, and represents a challenge for public health due to its virulence and ability to develop resistance to antimicrobials [1]. *S*. *aureus* is one of the leading causes of nosocomial bloodstream infections (BSI) in Brazil and is associated with high mortality (31%) [2]. Methicillin-resistant *S*. *aureus* (MRSA) isolates harbor the *mec*A gene, which is located into the staphylococcal cassette chromosome *mec* (SCC*mec*). Hospital-acquired MRSA (HA-MRSA) isolates traditionally carry SCC*mec* types II and III, while type IV is often found in community-acquired MRSA [3]. However, these boundaries have become blurred with epidemiological studies showing a change in circulating lineages within hospitals [3], [4].

There are several options for the treatment of MRSA infections including such as linezolid, tigecycline, daptomycin and ceftaroline, being the last one the active metabolite of ceftaroline fosamil. However, vancomycin has continued to be the primary treatment for the last fifty years [5]. Nevertheless there has been reports of isolates with reduced susceptibility to vancomycin, including both vancomycin-intermediate resistant (VISA) and heteroresistant *S*. *aureus* (hVISA)[6].

The relevance of heteroresistant isolates remains unclear. Some studies indicate that these isolates may be associated with persistent bacteremia and treatment failure and hVISA may be precursor of the VISA phenotype [7], [8]. The hVISA phenotype is currently not identified by routine laboratory procedures because only one in 10^−6^ cells grow in concentrations of vancomycin within the intermediate susceptibility range and they grow more slowly [7], [8]. Population analysis profile (PAP) test remains the gold standard method to detect hVISA. Other screening methods, such those based on Etest^®^ (Glycopeptides Resistance Detection—GRD and macromethod) and Brain Heart Infusion (BHI) agar, have also been used but performance is variable [9].

Despite a previous report of vancomycin resistant *S*. *aureus* (VRSA) isolated from a bloodstream infection in São Paulo in 2013 [10], hVISA [11] and VISA [12], [13] isolates appear to be uncommon in Brazil. We previously reported that 124 *S*. *aureus* isolates from patient with bloodstream infection (BSI) from two tertiary hospitals in Rio de Janeiro, between 2008 and 2009, were susceptible to vancomycin [14]. However, VISA and hVISA infections are reported worldwide and have been associated with poor patient outcomes [7], [8], [15]. In the present study, we characterized a collection of *S*. *aureus* isolates from BSIs from a Rio de Janeiro teaching hospital to determine the presence of VISA and hVISA isolates using 1) broth microdilution method (BMD) to determine vancomycin MIC; 2) BHI agar plates containing different vancomycin concentrations, Etest® methods (Etest® GRD and Etest^®^ macromethod) and population analysis profile/area under the curve (PAP-AUC); 3) BMD to test susceptibility to oxacillin, teicoplanin, linezolid, daptomycin, tigecycline and ceftaroline; 4) PCR for *mec*A gene detection and SCC*mec* typing; 5) pulsed-field gel electrophoresis (PFGE) and multilocus sequence typing (MLST) to assess the clonality and 6) medical charts to identify clinical aspects of the patients.

## Materials and Methods

### Clinical isolates, setting and ethics statement

This study was performed at the University Hospital Clementino Fraga Filho, a tertiary public teaching hospital in Rio de Janeiro, Brazil with about 70,000 patients-days per year. A retrospective study was conducted to evaluate phenotypic and molecular profile of *S*. *aureus* isolates from consecutive BSI from adults between February 2011 and December 2013. Only the first isolate of a BSI episode was included in the analysis. An episode was defined as an isolation of *S*. *aureus* in a blood culture with subsequent documentation of negative blood cultures, clinical improvement, and antistaphylococcal therapy. The study was approved by Human Research Ethics Committee of the University Hospital Clementino Fraga Filho (number 976.427). Patient demographics, Charlson Comorbidity Index score [16], classification of the BSI episode [17], treatment, length of stay and discharge were collected.

All blood cultures were processed using BacT/ALERT^®^ (BioMerieux Inc., Durham, NC, USA). Bacterial identification was carried out by the automated VITEK 2^®^ system (BioMerieux, Durham, NC, USA). Identification of bacteria was confirmed using: Gram staining, catalase and coagulase production, and evaluation of 0.04 U bacitracin resistance by disk-diffusion [18].

### Antimicrobial susceptibility tests and SCCmec typing

Susceptibility to methicillin was determined by cefoxitin (CECON, São Paulo, Brazil) disk diffusion test according to the CLSI [19]. MICs were determined by BMD, using fresh cation-adjusted Muller-Hinton broth (CAMHB) for oxacillin, vancomycin, teicoplanin, linezolid, daptomycin, tigecycline (Sigma-Aldrich Chemical Company, St Louis, MO, USA) and ceftaroline (donated by AstraZeneca Pharmaceuticals, Schaumburg, IL, USA) [19]. CAMHB was supplemented to 50 μg/mL calcium for the daptomycin assay [19]. The CLSI interpretative breakpoints were used for all antimicrobial agents except for tigecycline, for which EUCAST 2015 breakpoints [20] were used as the first does not establish a breakpoint for this antimicrobial. ATCC strains 25923 and 29213 were used as controls for the disk diffusion and MIC tests, respectively. The *mec*A gene detection and SCC*mec* typing were performed as previously described [21].

### Phenotypic tests for screening of hVISA

Isolates with an MIC of 2 mg/L by the BMD method were screened for hVISA. Six screening tests were used according to previous reports [9], [19], [22], [23]. Plates with BHI agar (BBL, Becton Dickson, MD) containing 3, 4, 6 mg/L of vancomycin (BHIa3, BHIa4 and BHIa6, respectively) were swabbed with 0.5 McFarland standard suspension (10^8^ CFU/mL) of *S*. *aureus* and incubated at 35 to 37°C for 48 h. Reduced susceptibility to vancomycin was defined as growth of one or more colonies at any of the three concentrations [19], [22], [23]. BHI agar plates containing 4 mg/L of vancomycin and 16 g/L of pancreatic digest of casein (Merck, Darmstadt, Germany) (BHI4ca) were inoculated using four 10 μL spots from a 0.5 McFarland standard inoculum, as previously described [9]. An isolate was considered to have reduced susceptibility if at least one spot had two or more colonies. All isolates were also screened for VISA using the BHIa3, BHIa4, BHI4ca and BHIa6.

Etest® GRD (bioMerieux) was performed using a suspension at 0.5 McFarland swabbed onto Muller-Hinton 5% sheep blood agar plates (Biocampo, Friburgo, Brazil) and incubated at 35 to 37°C. The vancomycin and teicoplanin Etest^®^ macromethod (bioMerieux) was performed using a 2.0 McFarland inoculum (6 x 10^8^ CFU/mL) of *S*. *aureus* on BHI agar plates. Isolates were considered hVISA when the MIC value was ≥ 8 mg/L for both, teicoplanin and vancomycin, or 12 mg/L for teicoplanin, after 48 h of incubation, regardless of vancomycin MIC [9]. *S*. *aureus* ATCC 29213 (MSSA), and Mu3 (hVISA) and Mu50 (VISA) were used in all screening tests as control strains [24].

### Population analysis profile to confirm hVISA and VISA isolates

MRSA isolates displaying at least one positive screening test result were confirmed to be hVISA by population analysis profile/area under the curve (PAP-AUC) [25]. Representative VISA isolates were also selected to be tested by PAP-AUC. Briefly, after 24 h of incubation on blood agar, colonies were suspended in saline and plated in BHI agar containing 0, 0.5, 1, 2, 3, 4 and 8 mg/L of vancomycin. After 48 h of bacterial growth at 35 to 37°C, bacterial colony counts (log_10_ numbers CFU/mL) were plotted against the vancomycin concentration. The graph obtained was used to calculate the area under the curve (AUC) for each isolate. A ratio between the AUC of the test isolate to the AUC of the reference strain Mu3 was calculated. The isolates were identified as hVISA or VISA if the ratio of the AUC was ≥ 0.9 or > 1.3, respectively.

### Pulsed-field gel electrophoresis and multilocus sequence typing

All isolates identified as VISA or hVISA were typed by pulsed-field gel electrophoresis (PFGE) after digestion of whole cell DNA with *Sma*I in a CHEF-DRIII system (Bio-Rad, Richmond, CA, USA), as previously described [26]. The PFGE fingerprints were compared by the unweighted pair-group method with arithmetic mean (UPGMA) clustering analysis, applying the Dice correlation coefficient. Isolates with four or fewer bands of difference and minimum of 80% similarity were designated as the same genotype [27]. The clonal profiles obtained were compared with those previously described for identification of national and international clones [3], [28]. Multilocus sequence typing (MLST) was performed to determine the sequence type of the isolate [29].

### Statistical analysis

Two tailed Fisher´s exact test were used to calculate *p-*values. A difference for which *p ≤* 0.05 was considered statistically significant.

## Results

One hundred and ten *S*. *aureus* isolates from consecutive BSIs were analyzed. Five patients presented two different episodes of BSI. Thirty one (28%) isolates were MRSA. Oxacillin MICs ranged from < 0.25 to > 256 mg/L, and the MIC_50_ and MIC_90_ values were 0.5 and 128 mg/L, respectively. Two (2.5%) of 79 MSSA were intermediate resistant to vancomycin compared with 4 (13%) of 31 MRSA (p = 0.052). Five MSSA isolates (6.3%) were non-susceptible to daptomycin compared with 11 (35.5%) MRSA isolates (p = 0.0003) (Table 1). All isolates were susceptible to linezolid, tigecycline and teicoplanin. Among the 31 MRSA isolates, 15 (48%) carried the SCC*mec* II and 16 carried the SCC*mec* IV. No VRSA isolate was detected.

**Table 1:** Antimicrobial susceptibility in mg/L determined by the broth microdilution method in 110 *Staphylococcus aureus* isolates from bloodstream infections.

Antimicrobial	MSSA (n = 79)				MRSA (n = 31)				*p*-value
	Range	MIC_50_	MIC_90_	% of non-susceptible isolates*	Range	MIC_50_	MIC_90_	% of non-susceptible isolates*	
**Vancomycin**	0.5–4	1	2	2.5	0.5–4	2	4	13	0.052
**Teicoplanin**	0.25–1	0.25	0.5	0	0.25–4	0.5	1	0	NA
**Linezolid**	0.25–4	2	4	0	1–4	2	2	0	NA
**Daptomycin**	0.25–2	1	1	6.3	0.5–4	1	4	35.5	0.0003
**Tigecycline**	0.125–0.5	0.25	0.5	0	0.125–0.5	0.25	0.5	0	NA
**Ceftaroline**	0.0625–0.5	0.125	0.25	0	0.25–2	0.5	1	3.2	0.29

MIC: minimal inhibitory concentration; NA: not applicable.

* % of non-susceptible isolates was determined according to the CLSI interpretation criteria; EUCAST breakpoints were used for tigecycline.

Six VISA isolates with vancomycin MIC of 4 mg/L by the BMD (isolates numbers 1579, 1582, 1616, 1698, 1638 and 1645) were detected and, four of them were MRSA (**Table 2**). Three of the 4 MRSA-VISA isolates with SCC*mec* II presenting the same PFGE profile were characterized as USA100 by PFGE and ST5/Clonal Complex (CC) 5 lineage by multilocus sequencing typing. The other MRSA carried the SCC*mec* IV and was USA800 by PFGE and ST3192/CC5. This new ST3192, described in this study, is a double locus variant (DLV) of ST5. Isolate number 1579 (USA100) was non-susceptible to ceftaroline by the BMD according to CLSI interpretative criteria (MIC of 2 mg/L) and, except for this isolate, all MRSA-VISA isolates were non-susceptible to daptomycin by CLSI interpretative criteria (MIC ≥ 2mg/L). The automated VITEK 2^®^ system (BioMérieux) failed to detect any of the six VISA isolates and E-test detected only 1.

**Table 2:** Microbiological characteristics of the six vancomycin-intermediate *Staphylococcus aureus* (VISA) isolates.

Isolate number	Vancomycin MIC (mg/L)			Broth microdilution MIC (mg/L)		Screening plates (48h of incubation)			Methicillin resistance/SCC*mec* type	PFGE pattern	Allelic profile (arc-aro-glp-gmk-pta-tpi-yqi genes)	ST	CC	Clonality
	E-test	Vitek 2	BMD	DAP ^a^	CPT ^b^	BHIa3	BHIa4	BHI4ca						
**1579**	2	2	4	1	2	+	-	+	MRSA/II	A	1-4-1-4-12-1-10	5	5	USA100
**1582**	1.5	2	4	2	1	+	-	-	MRSA/II	A	1-4-1-4-12-1-10	5	5	USA100
**1616**	1	2	4	4	1	+	+	+	MRSA/II	A	1-4-1-4-12-1-10	5	5	USA100
**1698**	4	1	4	4	0.5	+	-	-	MRSA/ IV	B1	1-233-1-8-12-1-10	3192	5	USA800
**1638**	1	1	4	1	0.125	-	-	-	MSSA	B2	1-4-1-4-12-1-10	5	5	USA800
**1645**	1	1	4	1	0.125	+	+	+	MSSA	C	3-1-1-8-1-1-1	188	1	ND

MIC: minimal inhibitory concentration; PFGE: pulsed-field gel electrophoresis; ST: sequence type; CC: clonal complex; BMD: broth microdilution; ND: not determined

^a^ Daptomycin—according to Clinical and Laboratory Standart Institute (CLSI), MIC value ≤ 1 mg/L is considered susceptible

^b^ Ceftaroline—according to CLSI, MICs ≤ 1 mg/L, 2 mg/L and ≥ 4 mg/L are classified as susceptible, intermediate or resistant isolates, respectively.

**Fig 1:**
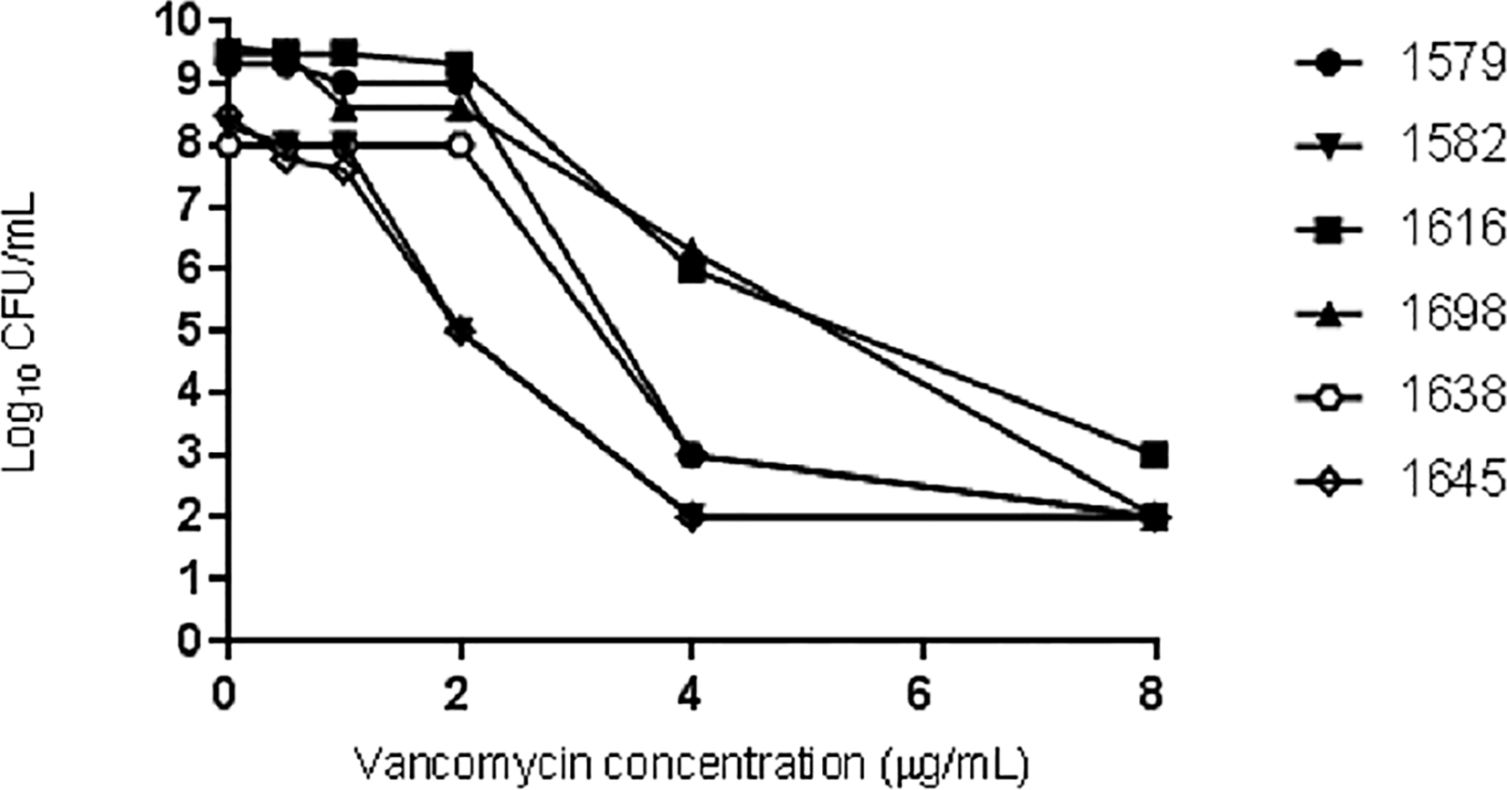
Growth curves of the six vancomycin-intermediate *S*. *aureus* isolates.

Among the six VISA isolates (MIC = 4 mg/L), five (83%) grew on BHIa3. Two (33%) of them also grew on BHIa4 and three (50%) on BHIa4ca media (**Table** 2). No VISA isolate was positive for the BHIa6 or Etest^®^ GRD tests. The Fig 1 shows the population analysis profile (PAP) of vancomycin among the six VISA isolates. The analyzed VISA isolates 1579, 1582, 1616, 1698, 1638, 1645 had a PAP-AUC ratio of 2.75,2.02, 3.57, 3.38, 2.52 and 2.00 respectively, and were confirmed as VISA by this method.

All patients with VISA isolates had significant co-morbidities and all 6 VISA BSIs were health care-associated (**Table** 3). Two patients with isolates of the US100/ST5 lineage acquired the infections in the ICU, were treated with vancomycin and died due to the VISA infection. Among the four patients who were discharged from the hospital, two had infections by MSSA isolates and were successfully treated with beta-lactams (**Table** 3). Four patients had undergone dialysis treatment, although not at the same unit, prior to diagnosis of the VISA infection (**Table** 3).

**Table 3:** Clinical characteristics of the six patients with bloodstream infections caused by vancomycin intermediate *Staphylococcus aureus* (VISA) isolates.

Isolate number	Cause of admission	Gender	Charlson score	Dialysis	Ward	Previous therapy	Treatment after bacterial isolation	Outcome (number of days from therapy to outcome); Type of VISA infection
**1579**	Community-acquired Pneumonia	M	6	Acute hemodialysis	ICU	amx + sul + azm// tzp // tei + tzp //tei //van + mem + flu	van + mem + flu	Death (6); VAP infection
**1582**	Bacteremia (VISA) due to dialysis	F	4	Peritonial dialysis	Nephrology	Amk	van + amk//van	Discharge (13); Bacteremia related to dialysis
**1616**	Liver transplantation	F	4	Acute hemodialysis	ICU	nor + cro // sul + flu + amx// van + pmb + mem	Van	Death (4); Bacteremia due to central vascular catheter
**1698**	Infection (VISA) after vascular surgery	M	3	No	Internal Medicine	van // sxt // fep// tei	tei // dap // sxt + dap // gen + dap// dap // dap + fep // cip	Discharge (59); Nosocomial osteomyelitis with endocarditis
**1638**	Cancer	F	6	No	Oncology	fep // fep + van	fep // fep + van // oxa	Discharge (36); Bacteremia due to peripheral vascular catheter
**1645**	Bacteremia due to hemodialysis	M	2	Chronic hemodialysis	Nephrology	Cip	cfz // oxa + amk// cfz	Discharge (16); Bacteremia due to central vascular catheter

M = male; F = female; ICU = intensive care unit; MRSA = Methicillin-resistant *Staphylococcus aureus;* VAP = Ventilator-associated pneumonia; amk = amikacin; cip = ciprofloxacin; mem = meropenem; pmb = polimixin B; tzp = piperacillin/tazobactam; van = vancomycin; amx = amoxicillin; sul = sulbactam; azm = azithromycin; tei = teicoplanin; flz = fluconazole; nor = norfloxacin; cro = ceftriaxone; fep = cefepime; sxt = trimethoprim-sulfamethoxazole; oxa = oxacillin; cfz = cefazolin; gen = gentamicin; dap = daptomycin; // = interruption of treatment; patients age ranged from 46 to 83 years old.

Twenty-five *S*. *aureus* isolates with a vancomycin MIC of 2 mg/L by the BMD method were screened for detection of hVISA and six of them were positive by at least one assay (BHI screening plates, Etest^®^ GRD or Etest^®^ macromethod) (**Table** 4). Only one (isolate 1594) of the two MRSA isolates was confirmed hVISA by the PAP-AUC method (**Table** 4). Isolate number 1594 was positive in BHIa3, BHIa4, BHIa4ca and Etest^®^ macromethod screening methods (**Table** 4) and showed reduced susceptibility to daptomycin (MIC of 2 mg/L).

**Table 4:** Microbiological characteristics of six *Staphylococcus aureus* isolates presenting vancomycin MIC of 2 mg/L with at least one screening test positive to detect heterogeneous vancomycin intermediate resistance (hVISA).

Isolatenumber	Methicillin resistance/SCC*mec* type	Broth microdilution test MIC (mg/L)^a^			Screening plates (48h of incubation)				Etest macro^b^ (μg/mL)		Etest GRD^b^ (μg/mL)		PAP-AUC ratio^c^	Interpretation (Clonality)
		TEI	OXA	DAP	BHIa3	BHIa4	BHI4ca	BHIa6	VAN	TEI	VAN	TEI		
**1594**	SCC*mec*II	0.5	128	2	+	+	+	-	3	16	1	3	1.15	hVISA (USA100/ST5/CC5)
**1636**	SCC*mec*IV	0.25	8	1	+	-	-	-	4	4	1	1.5	0.50	False positive
**1588**	MSSA	0.25	0.5	1	-	-	-	-	4	12	1	3	ND	NA
**1595**	MSSA	0.5	0.5	2	-	-	-	-	3	16	0.5	3	ND	NA
**1622**	MSSA	0.25	0.25	1	+	-	-	-	2	2	0.75	1.5	ND	NA
**1691**	MSSA	0.25	≤0.2	1	+	-	-	-	6	4	0.75	3	ND	NA

^a^ Broth microdilution test according to CLSI guideline was used to determine the minimum inhibitory concentration (MIC)

^b^ The isolate was presumable hVISA if vancomycin and teicoplanin MICs were 8 μg/ml or if teicoplanin MIC was 12 μg/ml regardless of the vancomycin MIC

^c^ PAP-AUC: Population analysis profile-area under the curve, PAP-AUC was conducted only for MRSA isolates. The isolate was identified as hVISA if the ratio of the AUC of the isolate test to that of the reference strain (Mu3) was ≥ 0.9; ND: not determined; NA: not aplicable; TEI: teicoplanin; OXA: oxacillin; DAP: daptomycin; BHIa3, BHIa4, BHIa6 and BHI4ca: Brain Heart Infusion (BHI) agar containing 3, 4, 6 mg/L of vancomycin and containing 4 mg/L of vancomycin plus 16 g/L of pancreatic digest, respectively.

The patient with hVISA (isolate 1594, **Table** 4), an 83 years old male, had a history of previous hospitalization in the cardiology ward due to cardiac arrhythmia and was admitted at the Intensive Care Unit (ICU) on February 2011 due to community-acquired pneumonia. He was treated with a combination of amoxicillin and sulbactam for three days and then cefepime and azithromycin for additional five days. A nasal swab culture was positive for MRSA. Nine days after admission, teicoplanin and cefepime treatment began when primary MRSA bacteremia was diagnosed. After six days the patient died due to the hVISA infection. The MRSA strain from the nasal swab culture was not available for genotyping.

## Discussion

Vancomycin is a drug-of-choice for treatment of MRSA infections [5]. However, clinical outcomes for VISA and hVISA infections are poor [7],[8]. Our results showed that VISA and hVISA isolates were from healthcare-associated BSIs and were predominantly from SCC*mec* type II/USA100/ST5 lineage. Non-susceptibility to daptomycin (MIC ≥ 2 mg/L) was common among VISA isolates and one isolate had intermediate susceptibility (MIC of 2 mg/L) to ceftaroline, a drug that had not yet been used in clinical practice in Brazil at the time the sample was isolated.

Previous reports from the United States have shown the emergence of VISA-type resistance in type II USA100/ST5/CC5 isolates [30], [31], which corresponds to the prevalent VISA clone in the present study. In the past, VISA Brazilian isolates were related to the endemic clone of the ST239/CC8 lineage [12], however we reported in a previous study, performed between 2004 and 2007, dissemination of the USA100 and other lineages in a military institution in Rio de Janeiro [3]. The hVISA isolated in the present study was also characterized as USA100/ST5/CC5, suggesting that it may be a precursor of VISA phenotype. Moreover, because the patient infected with hVISA in the present study was colonized with a MRSA isolate on admission prior to isolation of hVISA from blood, it is possible that colonizing MRSA was the source of the hVISA. However, this could not be confirmed because the colonizing MRSA was unavailable for genotyping. hVISA isolates of the SCC*mec* II USA100/ST5/CC5 lineage have been previously identified in Japan, Sweden, France, Poland, United Kingdom, USA and Norway [30]. Of note, most VRSA isolates described worldwide belong to the same CC5 [32]. Thus, the emergence of this clone in Brazil could presage future reports of VISA, hVISA and VRSA isolates in our health institutions.

In the present study, we detected 3% (1/31) of hVISA among MRSA isolates causing BSI belonging to the same CC5 of VISA isolates, which accounted for 5% (6/110) of the total isolates. A study in southern Brazil from 2009–2013 that analyzed 124 *S*. *aureus* isolates found 12 (9.7%) hVISA isolates, although no VISA isolate was detected. However, the study authors did not characterize the clonality of these isolates [11]. In a previous study conducted in the Northeast China [15] from 2007 to 2010 among 757 *S*. *aureus* clinical isolates the VISA rate was 0.5% and the majority of which were ST5/CC5 [15], which is consistent with our results.

It is known that some VISA clonal types, especially MRSA, can easily disseminate [33]. In the present study, the results showed that although not all patients were located at the same ward, or even hospitalized at the same time, a possible dissemination of the USA100/ST5 lineage may have occurred in the hospital, probably carried by the hospital professionals. As in our study [13], we found that four of the six patients with BSI caused by VISA isolates had undergone dialysis treatment before isolation of a VISA strain. Vancomycin is a key antibiotic for the treatment of gram-positive infections and this may select for the VISA phenotype [7], [33], [34]. Therefore, control and prevention of infections and judicious use of antimicrobials should be revised in the institution.

For identification of hVISA, the PAP-AUC remains the gold standard, but it is an expensive and labor-intensive technique. Various screening methods have been described [9], [11], [35] and the best screening test is yet to be defined. In the present study we used six different methods to screen hVISA among 25 isolates presenting MIC of 2 mg/L and six potential hVISA isolates were detected. Two MRSA that were potential hVISA isolates were tested by PAP-AUC and one was confirmed as vancomycin heteroresistant. The BHIa6 was unable to detect hVISA, and as shown previously, is not an effective screening tool [35]. On the other hand, agar screening is inexpensive and the addition of supplements like pancreatic digest of casein to BHIa4 could improvement detection of hVISA [9]. Satola *et al* [9] found 91% and 94% of sensitivity and specificity values, respectively for BHI4ca in determining hVISA after analysis of 140 MRSA clinical isolates presenting a vancomycin MIC of 2 mg/L. In the present study the BHI4ca assay was more sensitive than Etest^®^ GRD. Interestingly, among the VISA isolates (MIC = 4 mg/L) five (83%) grew on BHIa3 and three of them (50%) grew on BHI4ca (data not shown). Use of both media could help detect not only hVISA but also VISA isolates. However, other studies are necessary to confirm these findings.

We characterized a ceftaroline-intermediate *S*. *aureus* (CISA; MIC of 2 mg/L) isolate and the clinical aspects of the patient infected with this isolate. This CISA isolate was also a VISA. Ceftaroline was only recently approved in Brazil and this isolate pre-dates its use clinically. A study that analyzed 956 *S*. *aureus* clinical isolates from different Latin America countries in 2011 [36] found 16.4% of isolates with MIC of 2 mg/L for ceftaroline. A more recent study analyzing *S*. *aureus* isolates from respiratory tract and skin infections from different countries found in Brazilian hospitals MRSA with ceftaroline MIC of 2 mg/L, however all isolates were susceptible to vancomycin [37]. *S*. *aureus* strains with a ceftaroline MIC_90_ of 2 μg/mL have been identified from seven countries in the Asia-Pacific region [38]. These data show the importance of decreased susceptibility of new drugs and the need to continually evaluate the dynamic changes that occur in bacterial resistance.

A substantial number of *S*. *aureus* isolates in the present study showed non-susceptibility to daptomycin with MIC values of 2 mg/L (12 isolates; 11%) and 4 mg/L (4; 4%) by BMD. The majority of these isolates were SCC*mec* II, including some MRSA-VISA isolates. Association between the VISA phenotype and non-susceptibility to daptomycin has been demonstrated [39]. We recently reported a case report of a BSI caused by a daptomycin non-susceptible MRSA/VISA isolate belonging to the lineage USA100/ST5 [13], confirming the emergence of this phenotype among *S*. *aureus* isolates in our country.

In summary our study detected 7 (6.4%) VISA/hVISA Brazilian isolates from BSI and it is the first report of a hVISA isolate in the city of Rio de Janeiro. In addition, we reported the occurrence of a single CISA isolate. The test BHI4ca could be considered to predict the VISA and hVISA phenotypes. Four patients with VISA and one with hVISA isolates had undergone dialysis treatment prior to diagnosis of infection, suggesting possible dissemination of the SCC*mec* II/USA100/ST5 lineage comprising the VISA, hVISA and daptomycin non-susceptible *S*. *aureus* isolates, representing a change in the epidemiological profile of BSI in the hospital.
